# Migraine and risk of narcolepsy in children: A nationwide longitudinal study

**DOI:** 10.1371/journal.pone.0189231

**Published:** 2017-12-07

**Authors:** Chun-Pai Yang, Meng-Lun Hsieh, Jen-Huai Chiang, Hsing-Yi Chang, Vivian Chia-Rong Hsieh

**Affiliations:** 1 Departments of Neurology, Kuang Tien General Hospital, Taichung, Taiwan; 2 Department of Nutrition, Huang-Kuang University, Taichung, Taiwan; 3 Department of Medical Education, Taipei Veterans General Hospital, Taipei, Taiwan; 4 School of Chinese Medicine, College of Chinese Medicine, China Medical University, Taichung, Taiwan; 5 Management Office for Health Data, China Medical University Hospital, Taichung, Taiwan; 6 College of Medicine, China Medical University, Taichung, Taiwan; 7 Institute of Population Health Sciences, National Health Research Institutes, Zhunan, Taiwan; 8 Department of Health Services Administration, College of Public Health, China Medical University, Taichung, Taiwan; University of Rome Tor Vergata, ITALY

## Abstract

**Background:**

The association between migraine and narcolepsy remains controversial. We aim to investigate whether migraine is associated with an increased risk of developing narcolepsy in children.

**Methods:**

In this longitudinal study, nationwide medical-claims data of pediatric patients (0-17y) with migraine are identified using the National Health Insurance Research Database (NHIRD) between 1997 and 2010 in Taiwan. Two cohorts are selected: migraine cases (n = 8,923) and propensity score-matched non-migraine controls (n = 35,692). Children with previous history of narcolepsy or headache before the index date are excluded. Cohorts are followed until the end of 2012, their withdrawal from the NHI program, or incidence of narcolepsy (ICD-9-CM: 347). Cox proportional hazards regression models are used to estimate hazard ratios (HRs) and 95% confidence intervals of developing narcolepsy in children with migraine compared to their non-migraine controls.

**Results:**

A total of 13 incident cases with narcolepsy are observed during follow-up, with incidence rates of 0.1915 and 0.0278 per 1,000 person-years in migraine and non-migraine children, respectively. After a mean follow-up period of 4.68 and 5.04 years in the case and control cohort, respectively, the former exhibited a greater risk of developing narcolepsy compared to the latter (adjusted hazard ratio (aHR) = 5.30, 95% confidence interval (CI): 1.61, 17.4; *p* = 0.006). This finding persisted after controlling for potential confounders like baseline comorbidities and concurrent medication uptake, and in our analyses with migraine subtypes.

**Conclusions:**

Migraine is an independent risk factor for narcolepsy development in children. Further studies are needed to validate our findings and to explore the exact pathophysiological mechanisms linking migraine and narcolepsy.

## Introduction

The association between migraine and narcolepsy has been evaluated in several hospital-based studies and still remains controversial [1–3]. These studies adopted case-control cross-sectional study designs with relatively small sample sizes, which could not allow us to firmly establish a causal relationship between migraine and narcolepsy, or suggest a mechanism that could explain such relationship. Furthermore, as almost one-third of patients become symptomatic before reaching the age of 15, there is an increasing level of attention given on the development of narcolepsy early in life [4]. Unfortunately, whether migraine is associated with increased risk of narcolepsy in children has not been explored in a large-scale longitudinal study.

Migraine has been considered a disturbance in the brain of the subcortical aminergic sensory modulatory systems, in addition to hypothalamic, thalamic and brainstem structures [5,6]. Hypothalamic orexinergic neurons actively enhance waking promoting monoaminergic and cholinergic hypothalamic and brainstem neural networks [7]. The destruction of orexinergic system results in narcolepsy as well as altered pain processing including migraine [8]. Given that migraine may share with narcolepsy in distinct brain areas especially in hypothalamus and neurotransmitter systems or both, we hypothesize that migraine can be linked to narcolepsy, particularly in children whose been observed with relatively high incidence. Therefore, we conduct a retrospective population-based cohort study using the Taiwan National Health Insurance Research Database (NHIRD) to investigate whether migraine is associated with an elevated risk of developing narcolepsy in children less than 18 years of age.

## Materials and methods

### Data source

A population-based longitudinal study was designed for this investigation. Our data source was the Taiwan National Health Insurance Research Database (NHIRD), which is a large computerized medical claims-based database released by the National Health Insurance Administration (NHIA) and maintained by the National Health Research Institutes (NHRI) for research purposes.

The NHI is a compulsory health insurance program in the country, offering a comprehensive benefits package. It currently has a national coverage of over 99% and is contracted with 97% of the health services providers in Taiwan. Thus, the NHIRD compiles an ample set of medical-claims records from preventive screening to medication history that is valuable for studying the health and service utilization of the entire population. For this study, we specifically used NHIRD registry files for children (aged <18 years) who are enrolled under the NHI program. From 1996 to 2012, a random, national-representative sample of enrolled children less than 18 years old were selected (n = 2,861,326). Outpatient, inpatient and medication records of these selected children were obtained for subsequent analysis. All diagnoses and disease definitions in this study were specified using the International Classification of Diseases, Ninth Revision, Clinical Modification (ICD-9-CM) codes.

All data from the NHIRD has been electronically encrypted for confidentiality reasons. Before its public release, all data with personal identification information, including patient identification number, birth date, gender, physician identification number and physician specialty, are all encrypted so that subjects can never be purposefully traced. The design and protocol of this study has been approved by the Research Ethics Committee at the China Medical University Hospital (CRREC-103-048(CR-2)).

### Study design & subject selection

**Fig 1:**
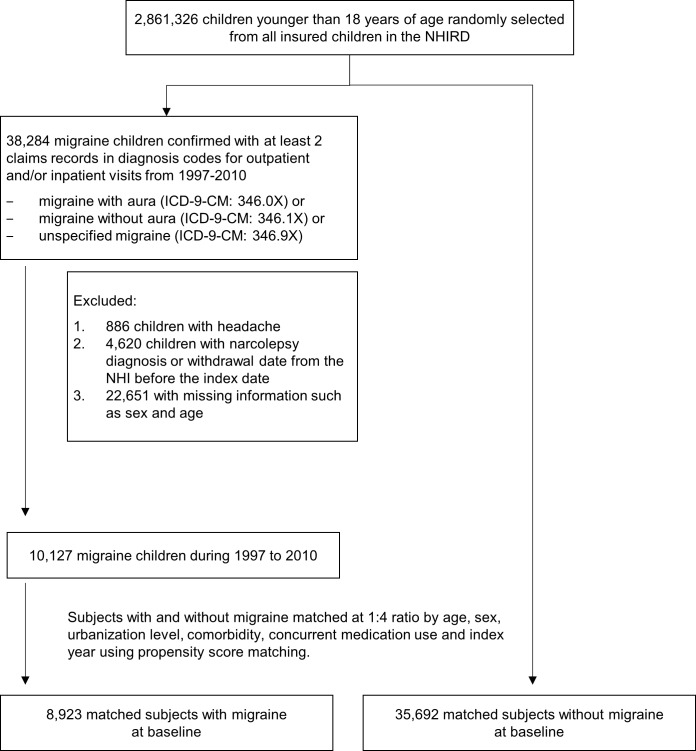
Subject selection flowchart.

For our study (migraine) cohort, we identified children aged less than 18 years from the random, national-representative sample who have newly diagnosed migraine between 1997 and 2010 (n = 38,284). We included different types of migraine including migraine with aura (ICD-9-CM: 346.0x), migraine without aura (ICD-9-CM: 346.1x) and unspecified migraine (ICD-9-CM: 346.9x). For ascertainment of cases, any identified subject should have at least two ambulatory care and/or inpatient visits with migraine diagnosis during the study period. We excluded those diagnosed with headache (ICD-9-CM: 346.2x; n = 886), missing information regarding subject’s sex and birthday, their date of narcolepsy diagnosis (n = 22,651), or those who had withdrawn from the NHI program before migraine diagnosis (n = 4,620) (Fig 1). A sum of 10,127 subjects resulted in the migraine cohort. For these children, their first date of migraine diagnosis became their index date of observation. In a similar manner, children without any migraine diagnosis from the random sample were identified during the same period of time to form the comparison (non-migraine) cohort. They were subsequently propensity-score-matched with the migraine children by age, sex, urbanization level, comorbidities, and index year at a 4:1 ratio (migraine: n = 8,923; non-migraine: n = 35,692). Analogous exclusion criteria were applied to the non-migraine cohort before matching.

### Outcome variable

Our primary outcome was the occurrence of narcolepsy (ICD-9-CM: 347), defined as the initial outpatient visit or hospital admission for narcolepsy during the follow-up period. Both cohorts were followed up until December 31 of 2012, their withdrawal from the NHI program, or the incidence of narcolepsy.

### Demographic and comorbidities profile

We considered baseline demographic variables including sex, age and urbanization level. Age was categorized into 3 groups: 0–5, 6–11 and 12–17 years old. For defining urbanization level of each subject’s area of residence, we used townships within which subjects enrolled their insurance as reference; four levels of urbanization (1 highest to 4 lowest) were classified based on a score calculated by incorporating variables indicating population density (number of people per squared kilometer), population ratio of different educational levels, elderly, agricultural workers and the number of physicians per 100,000 people [9]. Baseline comorbidities that could potentially influence our outcome were also considered. They included schizophrenia and organic psychosis (ICD-9-CM: 293–295), affective psychoses (ICD-9-CM: 296), neurotic disorders (ICD-9-CM: 300), depression (ICD-9-CM: 311), learning disability and developmental delay (ICD-9-CM: 315), mental retardation (ICD-9-CM: 317–319), central nervous system (CNS) infection (ICD-9-CM: 320–326), cerebral palsy (ICD-9-CM: 342–344), cerebral vascular disorder (ICD-9-CM:430–438), head injury with skull fracture (ICD-9-CM: 800–804, 850–854, 959.99), vision problems (ICD-9-CM: 36x-37x) and insomnia (ICD-9-CM: 307.41, 307.42, 307.49, 780.50, 780.52, 780.55, 780.56, 780.59). Effect of any medication containing nonsteroidal anti-inflammatory drug (NSAID), ergotamine, acetaminophen, triptan, antiepileptic, antipsychotic, and sedative-hypnotic that was used in these children during the study period was also considered.

### Statistical analysis

In our descriptive analysis, continuous data was expressed as means with standard deviation (SD) and categorical data were expressed as percentages (%). We compared baseline characteristics between children with and without migraine using standardized mean difference (SMD). SMD of less than 0.2 SD is indicative of negligible effect size in means or proportions of a propensity-score matched sample [10]. Incidence density rates were calculated for each cohort based on their incident events of narcolepsy and were expressed as per 1,000 person-years. Using log-rank test, the statistical difference in the cumulative incidence of narcolepsy between migraine and non-migraine cohorts was tested, and the Kaplan-Meier method was used to plot the survival probability. Both univariate and multivariate Cox proportional hazards regression models were performed to estimate hazard ratios (HRs) and their 95% confidence intervals (CIs) for the development of narcolepsy in the study cohort compared to the matched comparison cohort. All statistical tests were performed at the two-tailed significance level of 0.05. Analyses were carried out using the SAS statistical software (version 9.4 for Windows; SAS Institute, Inc., Cary, NC, USA).

## Results

After propensity-score matching, we selected 8,923 children with migraine and 35,692 control children without migraine for analysis. Table 1 describes the baseline characteristics of these identified subjects according to their migraine status (yes or no). As expected, subjects from both cohorts exhibited homogeneous post-match distributions for age, sex, urbanization level and comorbidities. Mean age was 11.58±3.88 and 11.02±3.94 years for migraine and non-migraine children, respectively, with a SMD of 0.145. In terms of baseline comorbidities and concurrent medication use, proportions of schizophrenia and organic psychosis, affective psychoses, neurotic disorders, depression, learning disability and developmental delay, mental retardation, CNS infection, cerebral palsy, cerebral vascular disorder, head injury with skull fracture, vision problems, insomnia, use of NSAIDs, ergotamine, acetaminophen, triptans and sedative-hypnotics are similar in migraine and non-migraine subjects. Concomitant use of antiepileptics and antipsychotics, however, were found with considerable differences (SMD>0.20). Thus, they were further adjusted for in the multivariate analyses. Among migraine children, most belonged to the unspecified migraine type (71.75%, n = 6,402). As a part of data exploration, we also found negligible SMD in the prevalence of other confounding conditions such as hyperactivity, infectious diseases of the upper respiratory tract, influenza and epilepsy between our matched children.

**Table 1:** Descriptive profile of the matched study and comparison cohorts.

Variable	Migraine				Standardized mean difference
	No (n = 35,692)		Yes (n = 8,923)		
	n	%	n	%	
**Follow time (mean, median) (years)**	5.04 (4.53)		4.68 (4.01)		
**Sex**					0.061
Girls	20842	58.39	4942	55.38	
Boys	14850	41.61	3981	44.62	
**Age (years)**					
0–5	4331	12.13	763	8.55	
6–11	15730	44.07	3818	42.79	
12–17	15631	43.79	4341	48.66	
Mean (SD^a^) (years)	11.02 (3.94)		11.58 (3.88)		0.145
**Urbanization level**					0.001
1 (highest)	10397	29.13	2659	29.80	
2	11118	31.15	2728	30.57	
3	6981	19.56	1661	18.61	
4 (lowest)	7196	20.16	1875	21.01	
**Comorbidities**					
Schizophrenia and organic psychosis	41	0.11	14	0.16	0.011
Affective psychoses	54	0.15	22	0.25	0.021
Neurotic disorders (anxiety)	784	2.20	210	2.35	0.011
Depression	40	0.11	13	0.15	0.009
Learning disability and developmental delay	831	2.33	260	2.91	0.037
Mental retardation (intellectual disability)	264	0.74	81	0.91	0.019
CNS infection	347	0.97	100	1.12	0.015
Cerebral palsy	143	0.40	45	0.50	0.015
Cerebral vascular disorder	159	0.45	45	0.50	0.009
Head injury with skull fracture	1854	5.19	513	5.75	0.024
Vision problems	30417	85.22	7195	80.63	0.122
Insomnia	759	2.13	234	2.62	0.033
**Concomitant medication use**					
NSAIDs	34175	95.75	8324	93.29	0.108
Ergotamine	5475	15.34	1418	15.89	0.015
Acetaminophen	34619	96.99	8414	94.3	0.132
Triptans	144	0.40	15	0.17	0.044
Antiepileptics	1062	2.98	981	10.99	0.319
Antipsychotics	4208	11.79	1782	19.97	0.225
Sedative-hypnotics	690	1.93	414	4.64	0.152
**Migraine Subtype**					
Migraine with aura	.	.	1049	11.76
Migraine without aura	.	.	1869	20.95	
Unspecified migraine	.	.	6402	71.75	

Chi-Square Test

^a^ t-test

SD: standard deviation; CNS: central nervous system; NSAIDs: nonsteroidal anti-inflammatory drugs

**Fig 2:**
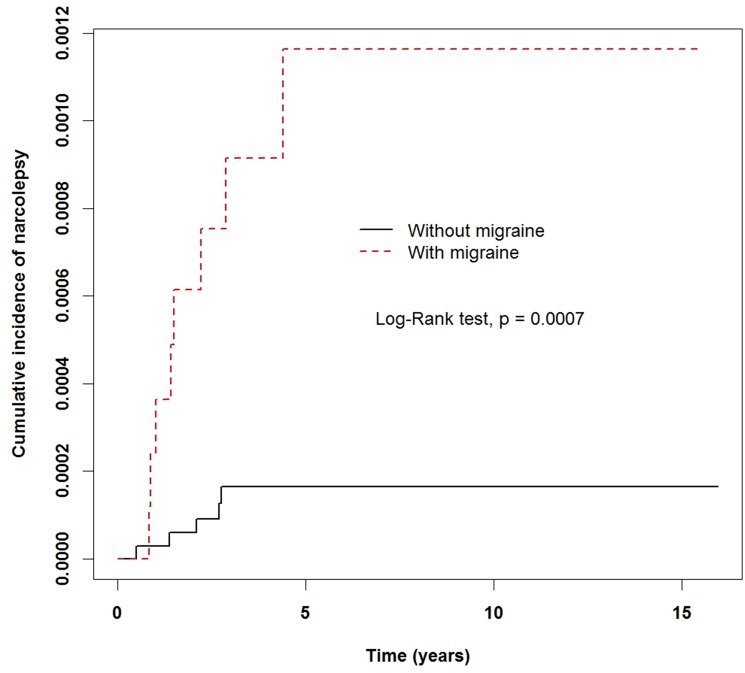
Cumulative incidence of narcolepsy in children diagnosed with migraine versus children without migraine. (dotted line) Incidence of narcolepsy in children of the migraine cohort in the observation period. (solid line) Incidence of narcolepsy in children of the non-migraine (reference) cohort.

A total of 13 subjects were newly diagnosed with narcolepsy during the follow-up period, with incidence rates of 0.1915 and 0.0278 per 1,000 person-years in the migraine and non-migraine cohorts, respectively. Table 2 displays results from univariate and multivariate Cox proportional hazards model analyses with the non-migraine group as the reference. Adjusted for the effects of sex, age, urbanization level, baseline comorbidities and concurrent medication use, a higher adjusted risk (aHR: 5.30, 95% CI: 1.61, 17.4, p = 0.006) of narcolepsy was observed in migraine children compared to non-migraine children. The Kaplan-Meier survival curve with log-rank test also showed higher cumulative incidence of narcolepsy in children with migraine (p = 0.0007) (Fig 2).

**Table 2:** Results from Cox proportional hazards model for the association between narcolepsy and migraine in pediatric subjects (hazard ratios and 95% confidence intervals).

Variable	Narcolepsy (n = 13)	Crude*			Adjusted^†^		
		HR	(95%CI)	p-value	HR	(95%CI)	p-value
**Migraine**							
No	5	1.00	reference		1.00	reference	
Yes	8	6.75	(2.21–20.6)	0.0008	5.30	(1.61–17.38)	0.0060
**Sex**							
Girls	6	1.00	reference		1.00	reference	
Boys	7	1.56	(0.52–4.64)	0.4236	1.51	(0.49–4.62)	0.4684
**Age (years)**							
0–5	0	-	-	-	-	-	-
6–11	4	1.00	reference		1.00	reference	
12–17	9	3.21	(0.97–10.6)	0.0558	2.51	(0.72–8.76)	0.1496
**Urbanization level**							
1 (highest)	6	1.00	reference		1.00	reference	
2	1	0.16	(0.02–1.29)	0.0848	0.18	(0.02–1.53)	0.1163
3	3	0.74	(0.19–2.97)	0.6728	0.93	(0.23–3.79)	0.9226
4 (lowest)	3	0.71	(0.18–2.84)	0.6282	0.87	(0.21–3.61)	0.8523
**Comorbidities**							
Schizophrenia and organic psychosis	0	-	-	-	-	-	-
Affective psychoses	0	-	-	-	-	-	-
Neurotic disorders (anxiety)	3	16.81	(4.61–61.3)	< .0001	8.33	(1.98–35.06)	0.0039
Depression	0	-	-	-	-	-	-
Learning disability and developmental delay	1	3.29	(0.43–25.3)	0.2521	2.76	(0.32–23.93)	0.3572
Mental retardation (intellectual disability)	0	-	-	-	-	-	-
CNS infection	0	-	-	-	-	-	-
Cerebral palsy	0	-	-	-	-	-	-
Cerebral vascular disorder	0	-	-	-	-	-	-
Head injury with skull fracture	1	1.57	(0.2–12.05)	0.6661	1.44	(0.18–11.59)	0.7325
Vision problems	12	2.23	(0.29–17.2)	0.4405	2.38	(0.30–18.95)	0.4130
Insomnia	2	10.58	(2.33–48.1)	0.0022	5.13	(0.99–26.64)	0.0518
**Concomitant medication use**							
NSAIDs	11	0.14	(0.03–0.63)	0.0109	0.27	(0.05–1.44)	0.1247
Ergotamine	4	2.19	(0.67–7.12)	0.1926	3.29	(0.94–11.44)	0.0617
Acetaminophen	12	0.19	(0.02–1.51)	0.117	0.46	(0.05–4.19)	0.4923
Triptans	0	-	-	-	-	-	-
Antiepileptics	3	5.85	(1.61–21.27)	0.0073	4.06	(0.99–16.61)	0.0511
Antipsychotics	1	0.49	(0.06–3.79)	0.4959	0.32	(0.04–2.55)	0.2813

NSAIDs–nonsteroidal anti-inflammatory drugs

Crude HR* represented relative hazard ratio

Adjusted HR^†^ represented adjusted hazard ratio: jointly adjusted for migraine, gender, age, insurance premium, urbanization level, comorbidities and concomitant medications in Cox proportional hazards regression.

Stratified by sex, the incidence rates of narcolepsy in girls and boys with migraine were 0.1382 and 0.2492 per 1,000 person-years, respectively; higher than those in the control cohort (0.0285 and 0.0268 per 1,000 person-years) (Table 3). In addition, migraine boys showed 6.68-fold (95% CI: 1.17, 38.1, p<0.05) higher adjusted risk of developing narcolepsy than the non-migraine cohort. Also observed with a statistically elevated risk of narcolepsy were migraine children belonging to age group of 12–17 years old (aHR: 4.20, 95% CI: 1.05, 16.7, p<0.05), those with listed comorbidities (aHR: 5.08, 95% CI: 1.56, 16.5, p<0.01), non-users of triptans (aHR: 5.08, 95% CI: 1.56, 16.5, p<0.01), or users of NSAIDs (aHR: 6.50, 95% CI: 1.81, 23.4, p<0.01) and acetaminophen (aHR: 4.87, 95% CI: 1.46, 16.3, p<0.01). Although regression models could not be analyzed for the use of antiepileptics or antipsychotics due to zero count in cells for controls, we observed that there was higher narcolepsy incidence in the migraine group compared to the non-migraine group: 3 events in cases vs. 0 event in controls who had concomitant antiepileptic use, and 1 event in cases vs. 0 event in controls who had concomitant antipsychotic use. Furthermore, among children without any concurrent antiepileptic or antipsychotic uptake, migraine children were observed with statistically higher risk of narcolepsy: aHR: 4.14, 95% CI: 1.16, 14.8, p<0.05 and aHR: 4.88, 95% CI: 1.46–16.3, p<0.01, respectively. Lastly, Table 4 gives the results of our sensitivity analysis with different migraine subtypes. The risk of narcolepsy is consistently higher in the migraine children when compared to non-migraine counterparts except for unspecified migraine: highest in migraine with aura (aHR: 9.03, 95% CI: 2.34, 34.8, p<0.01), followed by migraine without aura (aHR: 5.76, 95% CI: 1.48, 22.5, p<0.05).

**Table 3:** Incidence rates, hazard ratios and confidence intervals of narcolepsy for children patients with and without migraine stratified by demographic attribute, comorbidity and concomitant medication.

Variables	Migraine						Crude HR	Adjusted HR
	No			Yes				
	(n = 35,692)			(n = 8,923)				
	Event	Person years	IR	Event	Person years	IR	(95%CI)	(95%CI)
**Total**	5	179729	0.0278	8	41771	0.1915	6.75 (2.21–20.6)**	5.30 (1.61–17.38)**
**Sex**								
Girls	3	105086	0.0285	3	21704	0.1382	4.72 (0.95–23.4)	4.79 (0.91–25.09)
Boys	2	74644	0.0268	5	20067	0.2492	9.44 (1.83–48.7)**	6.68 (1.17–38.05)*
**Age (years)**								
0–5	0	35653	0	0	6769	0	-	-
6–11	1	97159	0.0103	3	22913	0.1309	12.58 (1.31–120.9)*	10.59 (0.99–113.66)
12–17	4	46917	0.0853	5	12077	0.4140	4.83 (1.30–17.9)*	4.20 (1.05–16.72)*
**Urbanization level**								
1 (highest)	3	50858	0.059	3	12751	0.2353	4.06 (0.82–20.1)	2.65 (0.48–14.77)
2	0	56128	0	1	12822	0.078	-	-
3	0	35947	0	3	8013	0.3744	-	-
4 (lowest)	2	36796	0.0544	1	8186	0.1222	2.02 (0.18–22.3)	2.27 (0.20–25.19)
**Comorbidities**								
No	0	30406	0	0	9348	0	-	-
Yes	5	149323	0.0335	8	32423	0.2467	7.09 (2.32–21.7)***	5.08 (1.56–16.54)**
**Concomitant medication use**								
NSAIDs								
No	1	3171	0.3154	1	1128	0.8862	2.91 (0.18–46.6)	2.08 (0.12–36.85)
Yes	4	176558	0.0227	7	40643	0.1722	7.48 (2.19–25.6)**	6.50 (1.81–23.38)**
Ergotamine								
No	5	146263	0.0342	4	34944	0.1145	3.36 (0.9–12.5)	3.20 (0.82–12.45)
Yes	0	33466	0	4	6827	0.5859	-	-
Acetaminophen								
No	0	1935	0	1	799	1.2522	-	-
Yes	5	177794	0.0281	7	40973	0.1708	5.96 (1.89–18.8)**	4.87 (1.46–16.26)*
Triptans								
No	5	178406	0.028	8	41673	0.192	6.73 (2.2–20.6)***	5.08 (1.56–16.54)**
Yes	0	1324	0	0	98	0	-	-
Antiepileptics								
No	5	172122	0.0290	5	36270	0.1379	4.63 (1.34–15.99)*	4.14 (1.16–14.82)*
Yes	0	7607	0	3	5501	0.5454	-	-
Antipsychotics								
No	5	154224	0.0324	7	32062	0.2183	6.59 (2.09–20.75)**	4.88 (1.46–16.31)**
Yes	0	25506	0	1	9709	0.1030	-	-

Abbreviation: IR, incidence rates, per 1,000 person-years; HR, hazard ratio; CI, confidence interval

Adjusted HR: adjusted for migraine, sex, age, insurance premium, urbanization level, comorbidities and drug used in Cox proportional hazard regression.

*:<0.05

**:<0.01

*** p<0.001

**Table 4:** Incidence rates and hazard ratios of narcolepsy in different migraine subtypes.

Migraine type	Event no.	Person-years	Incidence rate per 1000 person-years	Crude HR (95% CI)	Adjusted HR (95% CI)
**Migraine with aura (346.0x)**					
No	10	216430	0.05	1.00 (reference)	1.00 (reference)
Yes	3	5071	0.59	12.9 (3.55–46.9)***	9.03 (2.34–34.82)**
**Migraine without aura (346.1x)**					
No	10	213679	0.05	1.00 (reference)	1.00 (reference)
Yes	3	7821	0.38	7.39 (2.03–28.9)**	5.76 (1.48–22.45)*
**Unspecified migraine (346.9x)**					
No	8	190672	0.04	1.00 (reference)	1.00 (reference)
Yes	5	30828	0.16	3.88 (1.27–11.9)*	2.81 (0.85–9.22)

Abbreviation: HR, hazard ratio; CI, confidence interval

Adjusted for adjusted for migraine, sex, age, insurance premium, urbanization level, comorbidities and drug used in Cox proportional hazard regression.

*:<0.05

**:<0.01

*** p<0.001

## Discussion

This nationwide, population-based cohort study demonstrated that migraine in children less than 18 years of age was associated with a 5.30-fold increased risk of narcolepsy. This association was still present after controlling for the effects of potential confounders such as demographic characteristics, comorbidities and concurrent medication uptake. Furthermore, the elevated risk in migraine children was consistently detected in migraine with aura and migraine without aura subtypes, indicating that the study results were robust.

The major strength of the present study was the use of a nationwide population-based database, which enrolled a large sample size and appropriate observation period, thus providing sufficient power to delineate the differences between the two study cohorts. Moreover, a propensity score-matched comparison cohort was used to eliminate possibilities of subject selection bias and the influence of medical care-seeking behavior, thus reinforcing our conclusions that migraine increased the risk of narcolepsy in children. Although not many population-based estimates of narcolepsy in children are available, a European study has indicated an incidence rate of 0.83 out of 100,000 person-years in children between 5 and 19 years of age [11]. Here we observe an incidence rate of 2.78 per 100,000 (or 0.0278 per 1,000) person-years in the control cohort <18 years of age, which is higher than the European study estimate. However, this can be attributed to the fact that our pediatric sample is inclusive of children younger than 5 years old (i.e. lower incidence), or that the baseline properties have been slightly altered after matching with our study sample. Additionally, our study results are further validated since a statistically significant higher hazard ratio of narcolepsy is shown in the migraine children despite our comparatively higher observed incidence in the reference cohort. Occurrence of infectious events (infectious diseases of the upper respiratory tract and influenza) or other illnesses such as hyperactivity and epilepsy which could be capable of triggering narcolepsy onset were also found analogous between our two study cohorts, further confirming the relatively increased risk of narcolepsy attributed to migraine. Still, even after controlled for the effects of other central nervous system conditions like schizophrenia and organic psychosis, affective psychoses, neurotic disorders and others in the analyses, we cannot rule out the fact that these conditions could associate with a narcoleptic syndrome not confounded with idiopathic narcolepsy.

The association between migraine and narcolepsy was inconclusive. In the first hospital-based observational study, migraine showed a 2-fold to 4-fold increase in patients with narcolepsy [2]. This data supported their previous findings that half of narcoleptic patients reported migraine in a clinical sample. Nevertheless, a multi-centre case-control study with 96 narcoleptics did not find a significant higher prevalence of narcolepsy with migraine [3]. In contrast to these small sample size and case-control study design, our study included a relatively large number of population-based migraine pediatric cases, and a retrospective cohort study design with a sufficiently long follow-up period. Moreover, we focused on pediatric migraine cases, not adults like those addressed in previous studies [2,3]. Thus, our results support the emerging notion that migraine is associated with the risk of narcolepsy development in children. This association suggests a plausible pathophysiological mechanism that needs to be further investigated.

The exact pathophysiologic mechanisms linking migraine and narcolepsy are unknown. Loss of orexinergic neurons in the hypothalamus has been considered playing a causative role in narcolepsy with cataplexy. There is evidence that dysfunctional hypothalamic activity contributes to narcolepsy as well as altered pain processing via hypothalamic orexinergic neurons, while hypothalamus has been demonstrated in functional and structural brain imaging for migraine [12–15]. On other aspects, hypothalamic orexinergic neurons can stimulate neurons in the periaqueductal grey (PAG), locus coeruleus, and the raphe nuclei which inhibit antinociceptive activity in the TNC [13,14]. Due to the aforementioned relations, it is possible that hypothalamic orexinergic system is both involved in trigeminovascular nociception and narcolepsy developing that predispose some patients to migraine and narcolepsy. Clinical evidence for the role of hypothalamic orexinergic system in the modulation of migraine has not yet clearly identified and warrants further research.

Nevertheless, our results need to be interpreted with caution as several limitations are associated with the present study. First, the diagnoses of migraine and narcolepsy were entirely identified on the basis of ICD-9-CM codes, and therefore, were dependent on the diagnostic accuracy of the NHIRD database. These data may be less accurate than those diagnosed made in a prospective setting through standardized procedures, which may be an inherent weakness for all database research. The NHIA in Taiwan samples the medical charts routinely and has made every effort to verify the accuracy of the diagnoses in the database from every contracted medical institution [16]. Ideally, the diagnosis of narcolepsy should be based on the International Classification of Sleep Disorders (ICSD) 2 or 3 criteria which include results from diagnostic tests like multiple sleep latency testing (MSLT) and polysomnography (PSG). However, from our data, a lot of children with narcolepsy may not be diagnosed with the ICSD-2 or -3 diagnostic criteria as majority of data traces back to a period before ICSD-2. A small number of cases are detected even with a nationally representative sample in our study period from 1997 to 2010, suggesting that this disorder did not exhibit much medical attention previously. However, since narcolepsy is a rare disease, with the diagnosis large restricted to neurologists and sleep subspecialists rather than general physicians, its diagnostic accuracy should be failsafe. Furthermore, since many clinicians were unfamiliar with this disorder, narcolepsy remained under-diagnosed, potentially leading to underestimation of the risk of narcolepsy in the migraine cohort. Even so, there still exists the possibility that there are differential diagnoses of narcolepsy in the pediatric population such as delayed circadian phase syndrome or attention deficit hyperactivity disorder. In addition, the diagnosis of migraine was validated previously [17]. Nevertheless, a more definite diagnosis of migraine as a primary neurological disorder (versus unspecified migraine; with and without aura) is required to solidify the association between migraine and narcolepsy. Second, headache could potentially be a sign of sleepiness in children. But because we suspected potential confounding exerted by headache, patients with headache (ICD-9-CM: 346.2x; n = 886) were excluded in the selection process, which could lead to underestimation of results. Also, the frequency and severity of narcoleptic or migraine symptoms could not be precisely extracted from ICD codes, and this prevented further analysis. Third, patients in our migraine cohort were considered “active” migraineurs. Patients whose migraine became inactive may have been excluded from the migraine cohort and misclassified as controls. Under these conditions, the risk of developing narcolepsy among patients with migraine might have been underestimated. Fourth, the NHIRD is an administrative database which lacks thorough clinical data, such as disease onset and laboratory results. As a result, our results merely showed the increased associations of narcolepsy in patients with migraine, which may not allow a causal relationship to be established. Finally, most of our study subjects were ethnic Chinese from Taiwan, and the generalizability of our results to other ethnic groups needs to be further confirmed.

## Conclusion

This population-based longitudinal study provides evidence that migraine diagnosis is associated with an increased risk of narcolepsy in children. The higher narcolepsy incidence in the pediatric migraine population may indicate a common pathway of pathophysiology relevant to both disorders. Further research is warranted to describe the exact pathophysiological mechanisms linking migraine and narcolepsy.

## Public health relevance

- The association between migraine and narcolepsy in children has not been explored in a large-scale longitudinal study.
- As almost one-third of migraine patients become symptomatic before reaching the age of 15, there is an increasing level of attention given on the development of narcolepsy early in life.
- Using a nationwide population-based study approach, our results support the emerging notion that migraine is associated with the risk of developing narcolepsy in children.
- This derived association suggests a plausible pathophysiological mechanism that needs to be further investigated.
